# Parenting by Lying

**DOI:** 10.1177/09637214231206095

**Published:** 2023-12-05

**Authors:** Peipei Setoh, Petrina Hui Xian Low, Gail D. Heyman, Kang Lee

**Affiliations:** 1 Psychology, School of Social Sciences, Nanyang Technological University; 2 Department of Psychology, University of California, San Diego; 3 Department of Applied Psychology and Human Development, University of Toronto

**Keywords:** parenting by lying, parenting lies, parental lying, lying, parenting, socialization, honesty, dishonesty

## Abstract

Parenting by lying is a practice in which parents lie to their children to influence their emotions or behavior. Recently, researchers have tried to document the nature of this phenomenon and to understand its causes and consequences. The present research provides an overview of the research in the emerging field, describes some key theoretical and methodological challenges in studying this topic, and proposes a theoretical framework for understanding parenting by lying and for guiding future research to advance our knowledge about this understudied parenting practice.

Honesty is a highly endorsed virtue by parents around the world (Evans & Lee, 2022). Even though parents often preach to their children about the importance of being honest (Heyman et al., 2009), recent research suggests that it is common for parents to lie to their children. This apparent contradiction has the potential to present challenges to children as they navigate questions of when (if ever) dishonesty is appropriate.

The present article focuses on one type of parental dishonesty: when parents lie to their children to try to influence them. This practice, which has been referred to as *parenting by lying* (Heyman et al., 2013), was first described by Brown (2002) through ethnographic observation and first empirically investigated by Heyman et al. (2009). Subsequently, it has been documented in many countries, including the United States, Canada, China (Heyman et al., 2009, 2013; Liu & Wei, 2020; Santos et al., 2017), Singapore (Setoh et al., 2022), and Turkey (Jackson et al., 2021). This type of lying is of particular interest because it raises important psychological questions surrounding the intentions, mechanisms, and outcomes of such parental behavior.

## What Counts as Parenting by Lying?

Parenting by lying is a subset of the types of lying parents engage in. It is defined as verbal statements made by parents with an intention to deceive their children so as to influence them (Chisholm & Feehan, 1977; Evans & Lee, 2022). One of the most straightforward cases of parenting by lying is when parents lie to their children for behavioral compliance (Heyman et al., 2013; Kok et al., 2023). For example, parents may lie to their children by using false threats (e.g., “If you continue to mistreat your sister, I will call the police to put you in jail”; “If you don’t finish your rice, you will grow up to marry someone with pimples all over his face!”) and false promises (e.g., “If you finish your homework, I will take you to Disneyland”).

Parenting by lying can also be used to try to influence children’s feelings (Heyman et al., 2013; Setoh et al., 2022). For example, parents might say “That was beautiful piano playing” when they thought the playing was terrible and that a pet that died “went to live on a farm” to make a child less sad. Parents may also endorse imaginary beings they do not believe in, such as the Tooth Fairy, Easter Bunny, or Santa Claus, because they believe it will make their children’s childhood special. However, not all such lies are solely to influence children; for instance, parents might tell their children about Santa Claus to fit into their community or to recall fond memories of their own childhood.

The example about Santa Claus illustrates the intricate methodological and theoretical challenges associated with studying parenting by lying. These lies are characterized by the underlying intentions, yet researchers often struggle to access or discern parents’ true motives. At times, parents themselves may not recall or may be reluctant to acknowledge the real reasons behind their falsehoods. For instance, a parent might claim to be entertaining a child with whimsical answers like “The sky is blue because it’s the birds’ favorite color!” when the real intention might be to avoid the embarrassment of not knowing the answer. The situation is further complicated by the potential for differing perceptions of intentions between the teller and receiver of a lie (Bok, 1978) and the possibility that some seemingly false statements are actually metaphors or acts of pretend play. An example is a parent who tells her young child that she has eyes in the back of her head to see misbehavior, which she claimed that the child later recognized as a metaphor (Heyman et al., 2013). These nuances highlight the challenges of determining what counts as parenting by lying.

Most of the current research on parenting by lying has focused on lies concerning relatively mundane topics. However, parents also lie about more serious matters, such as medical conditions and drug abuse, where the stakes can be higher and the intentions more complex (Gingo et al., 2020). For instance, if parents falsely claim to their children that they never used drugs, this lie might be considered parenting by lying if the intention is to reinforce warnings against drug use. However, if the lie stems from a desire to protect personal privacy rather than to influence the child, it would not be considered parenting by lying. It will be critical for future research to examine these kinds of consequential lies.

## How Prevalent Is Parenting by Lying?

Parenting by lying appears to be highly common across the globe according to studies conducted across diverse populations. Most research on this subject has relied on retrospective parent-report methods or retrospective self-report methods, wherein adults are asked about their parents’ behaviors. For example, Heyman and colleagues (2013) found, using parent-report methods, that approximately 78% of American parents and 98% of Chinese parents engaged in parenting by lying. Interestingly, parents who perceived themselves as strongly committed to teaching their children that lying is wrong were just as likely as other parents to admit to this practice (Heyman et al., 2009). However, not all parents who lie do so regularly. Cargill and Curtis (2017) found that although 90% of young American adults reported that their parents had lied to them, only a small percentage (5%) stated that their parents lied often.

One important question for researchers working on parenting by lying is whether children are capable of detecting and reporting on the parenting by lying behavior of their parents. One study suggests they may be at least by early adolescence: Low et al. (2023) surveyed 11- and 12-year-olds and their parents independently about current experiences with parenting by lying and found consistency between parent and child reports.

In considering these findings, it is critical to acknowledge the methodological limitations. When people are retrospectively asked about lies they told, they will not necessarily be able to accurately remember what they are reporting on. Regardless of whether reports are done retrospectively or concurrently, people may be motivated to describe events in self-serving ways or fail to report events that they think will create a negative impression.

## What Factors Are Predictive of Parenting by Lying?

Several studies have examined the correlates of parenting by lying. This research suggests that many factors appear to be unrelated, such as children’s gender, political ideology, and number of children in the family (Heyman et al., 2009, 2013). There is mixed evidence concerning whether parents’ reported emphasis on honesty is related, with some findings suggesting that it is predictive of parenting by lying (Isik et al., 2023) and others suggesting that it may not be (Heyman et al., 2009).

Gingo et al. (2020) found that parents endorse parenting by lying to prevent risky behaviors less as children grow older to allow them to make prudential decisions on their own for the development of autonomy. These findings raise the possibility that the age of children may be predictive of the likelihood that parents will engage in certain forms of parenting by lying.

There is evidence that parental faith-based practices are negatively associated with parental lying: The more parents endorsed faith-based practices, the less likely they were engaged in parenting by lying to influence their children’s behavior (Setoh et al., 2022). However, this negative correlation may be due to self-presentation biases (Chung & Monroe, 2003).

There may also be cultural differences in parenting by lying. In line with this possibility, Heyman and colleagues (2009) found that Asian Americans were more likely to report and endorse parents’ lying to influence children’s behavior than were European Americans. Additional research conducted with Turkish college students found that almost all (96%) reported that their parents engaged in this practice (Jackson et al., 2021). These rates are comparable to reports of Asian participants (100%; Setoh et al., 2022) but higher than those of European Americans (78%; Heyman et al., 2013).

## Is Parenting by Lying Harmless?

Some researchers have raised concerns about the potential negative consequences of parenting by lying. For instance, it has been posited that this practice might inadvertently promote dishonesty in children (Santos et al., 2017). Such a possibility is aligned with social learning theory, which suggests that children may learn from the kinds of behaviors they see their parents model (Bandura, 1977). Additionally, other scholars have suggested that parenting by lying could undermine parent-child attachment (i.e., quality of the affectional bond between parent and child), creating disruptions or mistrust (Dodd & Malm, 2021; Liu & Wei, 2020).

These possibilities are corroborated with experimental evidence suggesting that when individuals lie to young children, they may be perceived as less trustworthy (Michaelson & Munakata, 2016), leading children to lie more to them in return (Hays & Carver, 2014). However, these findings were based on interactions with unfamiliar experimenters, and the dynamics may differ in a parent-child relationship. Additionally, these experiments assessed only immediate reactions to lying. The long-term effects of parenting by lying likely develop gradually in the context of the emerging parent-child relationship.

Studies have relied on correlational analyses to explore the outcomes of parenting by lying (Dodd & Malm, 2021; Santos et al., 2017; Setoh et al., 2020). For example, in Setoh et al.’s (2020) study, adults reported on the parenting by lying they experienced as children and their current deceptive behaviors and psychosocial adjustment. They found a positive correlation between recalled instances of parenting by lying and their propensity to lie to their parents in adulthood. They also found a negative association between reported levels of parenting by lying in childhood and current psychosocial maladjustment, such as externalizing problems (e.g., aggression, conduct problems) and internalizing problems (e.g., social withdrawal, anxiety, and depression). Further analysis suggested that the relationship between remembered exposure to parenting by lying and adult psychosocial outcomes might be mediated by the inclination of adults to lie to their parents. These findings underscore the complexity of the relationship between parenting by lying and its potential long-term effects on children’s behavior and well-being.

There have also been a small number of studies in which parenting by lying was assessed in childhood. In one such study, Low et al. (2023) tested a sample of Singaporean parents and their 11- and 12-year-old children. In line with the retrospective findings from young adults, Low et al. found that children’s reports of exposure to parenting by lying were positively correlated with their lying to their parents and negatively correlated with children’s psychosocial adjustment through the pathways of lying to parents and attachment to parents. In another such study, Jackson (2021) assessed parenting by lying among 6-to-12-year-old Canadian children. She found that greater exposure to parenting by lying was positively associated with both parent reports of children’s lying and children’s actual lying behavior observed in the laboratory.

Although recent studies incorporating concurrent reports of children’s exposure to parenting by lying and laboratory measures of lying have helped substantiate the correlation between parenting by lying and negative outcomes, these findings should be interpreted with caution. The studies are correlational, and many variables, such as child temperament, might influence these relationships. It is also possible that parenting by lying merely serves as a proxy for other parental qualities, such as poor self-control, that are driving these effects. Furthermore, the current measures need to be refined to be able to look at parenting by lying in a more nuanced way (e.g., separating lies to protect children’s feelings from lies intended to induce fear or compliance).

## A Theoretical Framework of Parenting by Lying

**Fig. 1.**
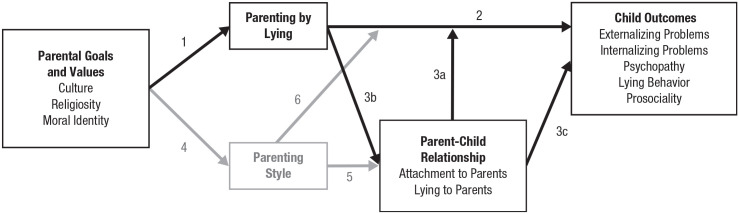
An integrative model of parenting by lying. The figure illustrates factors that have been studied with the practice of parenting by lying, mapping onto Darling and Steinberg’s (1993) original integrative model of parenting. Paths in solid black lines indicate theoretical links that have been supported by empirical evidence. Paths and variables in gray indicate theoretical links that have not been investigated with empirical research.

What parents do can have far-reaching and long-lasting implications on children’s well-being and development (Smetana, 2017). Parents use various methods to achieve their parenting goals. Parenting by lying represents one such method, and it should be viewed within the wider repertoire of parenting practices. We propose that one fruitful way to do this is to situate parenting by lying in Darling and Steinberg’s (1993) integrative model of parenting (Fig. 1). This model suggests that parental goals and values play an important role in influencing parenting practices and parenting styles. Parenting practices, in turn, directly impact children’s developmental outcomes. Parenting style serves a dual function: It moderates the link between parenting practices and children’s outcomes and influences the parent-child relationship.

With respect to aligning parenting by lying research with this theoretical model, parental goals and values that motivate parenting practices in general should play an important role in affecting whether parents engage in parenting by lying (Fig. 1, path 1). This framework suggests that it will be important to examine parenting goals and values (e.g., emphasis on achievement, discipline, or social competence) and specific parenting by lying practices. For example, a parent who highly values compliance may be more likely to lie to a child to promote compliance, which may be exacerbated by children’s tendencies to resist compliance and a lack of social support in achieving these goals in other ways.

The link between parenting by lying and children’s outcomes (Fig. 1, path 2) has been supported by the existing evidence. It has been found that exposure to parenting by lying was significantly associated with poorer psychosocial outcomes, such as externalizing problems, internalizing problems, psychopathy, lying behavior, and less prosociality (e.g., Dodd & Malm, 2021; Jackson et al., 2021; Low et al., 2023).

Darling and Steinberg (1993) highlighted the importance of parent-child relationships in moderating the pathway between parenting practices and child outcomes (Fig. 1, path 3a). In this regard, parent-child attachment has been shown to act as a buffer against the negative outcomes associated with parenting by lying (Liu & Wei, 2020). However, this is the only study to date that investigates a moderator of the relationship between parenting by lying and child outcomes, and other potential moderators (e.g., belief in parenting lies) should be investigated in the future.

New findings suggest that there are additional pathways of mediation between parenting by lying and child outcomes that have not been proposed by Darling and Steinberg. First, parenting by lying may lead to poorer parent-child relationships (Fig. 1, path 3b). Specifically, exposure to parenting by lying is associated with greater lying to parents (Santos et al., 2017; Setoh et al., 2020), poorer attachment to parents (Dodd & Malm, 2021; Low et al., 2023), and lower satisfaction with the parent-child relationship (Cargill & Curtis, 2017). Second, lying to parents and attachment to parents are in turn associated with child outcomes such as externalizing problems, internalizing problems, psychopathy, and prosociality (Fig. 1, path 3c; e.g., Jackson et al., 2021; Low et al., 2023).

Building on these findings, we propose a new mediation pathway between parenting by lying and children’s outcomes through the parent-child relationship (Fig. 1, paths 3b and 3c). The more that children are exposed to parenting by lying, the worse the parent-child relationship, which may then lead to poorer psychosocial adjustment. Although research established this mediation pathway initially only for lying to parents (Jackson et al., 2021; Santos et al., 2017; Setoh et al., 2020), recent evidence suggests this path also involves attachment to parents (Dodd & Malm, 2021; Low et al., 2023).

Darling and Steinberg’s (1993) model suggests that parental values and goals have another link to child outcomes via parenting styles, which has never been studied within the context of parenting by lying (Fig. 1, path 4). Also, nothing is known about how parenting styles are related to parent-child relationships in the context of parenting by lying (Fig. 1, path 5) or how they moderate the relationship between parenting by lying and child outcomes (Fig. 1, path 6). As parenting style is a crucial factor affecting parent-child relationships and child outcomes in general (e.g., Karavasilis et al., 2003; Kaufmann et al., 2000), future research can focus on these pathways to gain a fuller understanding of the impact of parenting by lying. For example, authoritarian parents may be more likely to engage in parenting by lying to gain compliance from their children than authoritative parents.

Overall, our proposed theoretical framework for parenting by lying builds on Darling and Steinberg’s (1993) integrative model of parenting. We have mapped the associations between parenting by lying and a range of child outcomes, either directly associated or mediated by the parent-child relationship. To develop a more robust theoretical framework, additional work is needed to investigate the relationship between parenting styles and parenting by lying.

## Future Research Directions

There are several promising avenues for future research, informed by the limitations previously identified and the existing theoretical framework. A first step is broadening the scope of research. This expansion includes not only documenting additional types of parenting by lying and their frequencies but also doing so in more cultures. We also need to explore the potential positive outcomes of certain types of parenting by lying. For example, it is possible parenting by lying motivated by benevolent parenting goals, such as sparing children’s feelings or maintaining a sense of magic, could be positively correlated with children’s self-esteem, cognitive skills, and creativity.

Our theoretical framework has also highlighted several major gaps in the current literature regarding predictors of parenting by lying. Such gaps need to be filled by future research to identify additional parenting values and goals that motivate the use of parenting by lying. In addition, we must examine such parental factors as parenting skills, mental health, childhood experiences, and moral socialization practices (Baumrind, 1971; Hart et al., 2020; Talwar et al., 2021). Environmental factors, such as household chaos or quality of parent relationships, should also be considered (Coldwell et al., 2006; Markowitz & Levine, 2021). Given the evolving parent-child relationship, the link between parenting lies and child factors may be bidirectional (e.g., parent-child relationship and lying within the dyad). Thus, future research should also consider such child factors as temperament, personality, and behavioral history (e.g., de Haan et al., 2012).

It is also important to examine some of the broader social dynamics surrounding parenting by lying, such as how children respond when discovering that their parents had lied and how parents respond when challenged about the truthfulness of their statements. Given that children learn about the social world through overheard conversation (Lane et al., 2020; Qin et al., 2021; Zhao et al., 2020), it will be important to examine children’s observations of parenting by lying that were not directed at them, such as when their parents lie to siblings. It will also be important to look closely at the factors parents consider when deciding whether to lie to their children. Heyman et al. (2013) found that some parents appeared to explicitly consider the costs and benefits of lying, such as one who reported lying to her child that a store had no candies because she thought doing so would save money and have no negative effects.

Finally, future research should diversify data collection methods and study designs. For example, future research may use longitudinal designs and statistical methods (e.g., crossed-lag panel or autoregressive latent trajectory models) to infer causal relationships between parenting by lying and various developmental outcomes. It will also be important to conduct intensive naturalistic observational research with the use of modern audio-video technologies (Vosoughi et al., 2012). Further, parenting interventions can be used to examine whether reducing parenting by lying leads to improved parent-child relationships and child outcomes. With the use of diverse research methodologies, we should be able to obtain an enriched picture of the parenting by lying practice and its short- and long-term effects.

## Conclusion

Existing research reveals that parenting by lying is a common practice. Correlational studies show that it is linked to short- or long-term negative psychosocial outcomes mediated by lying to parents by children and parent-child attachment. This work points to the need to examine whether these outcomes result from causal effects of parenting by lying and whether different forms of parenting by lying have different effects. The understanding of parenting by lying is currently in its infancy, with many unanswered questions. Future research should aim to provide a more comprehensive and nuanced scientific understanding of this phenomenon and offer parents evidence-based guidance on using this parenting practice.

## Recommended Reading

Gingo, M., Roded, A. D., & Turiel, E. (2020). (See References). An investigation into parents’ sociomoral justifications for telling parental lies.

Heyman, G. D., Hsu, A. S., Fu, G., & Lee, K. (2013). (See References). An introduction to parenting by lying and the most commonly used measure to report parental lies.

Liu, M., & Wei, H. (2020). (See References). An investigation of the effects of parenting by lying on internalizing problems in adolescence.

Santos, R. M., Zanette, S., Kwok, S. M., Heyman, G. D., & Lee, K. (2017). (See References). The first study to examine long-term consequences of parenting by lying in adulthood.

Talwar, V., Lavoie, J., & Crossman, A. M. (2021). (See References). The development of a measure to examine parents’ socialization of honesty in children, including parental modeling of lying.

## Transparency

*Action Editor:* Robert L. Goldstone

*Editor:* Robert L. Goldstone

*Author Contributions*

P. Setoh and P. H. X. Low are co-first authors. P. Setoh: conceptualization, supervision, writing – original draft, and writing – review and editing. P. H. X. Low: conceptualization, writing – original draft, and writing – review and editing. G. D. Heyman: conceptualization and writing – review and editing. K. Lee: conceptualization, supervision, writing – original draft, and writing – review and editing. All of the authors approved the final manuscript for submission.
